# Cancer stem cell related markers of radioresistance in head and neck squamous cell carcinoma

**DOI:** 10.18632/oncotarget.5417

**Published:** 2015-10-07

**Authors:** Ina Kurth, Linda Hein, Katrin Mäbert, Claudia Peitzsch, Lydia Koi, Monica Cojoc, Leoni Kunz-Schughart, Michael Baumann, Anna Dubrovska

**Affiliations:** ^1^ OncoRay-National Center for Radiation Research in Oncology, Faculty of Medicine and University Hospital Carl Gustav Carus, Technische Universität Dresden and Helmholtz-Zentrum Dresden-Rossendorf, Dresden, Germany; ^2^ Department of Radiation Oncology, Faculty of Medicine and University Hospital Carl Gustav Carus, Technische Universität Dresden, Dresden, Germany; ^3^ Helmholtz-Zentrum Dresden – Rossendorf, Institute of Radiooncology, Dresden, Germany; ^4^ German Cancer Consortium (DKTK), Dresden, Germany; ^5^ German Cancer Research Center (DKFZ), Heidelberg, Germany

**Keywords:** cancer stem cells, radioresistance, aldehyde dehydrogenase, head and neck squamous cell carcinoma (HNSCC)

## Abstract

Despite recent advances in understanding of the molecular pathogenesis and improvement of treatment techniques, locally advanced head and neck squamous cell carcinoma (HNSCC) remains associated with an unfavorable prognosis. Compelling evidence suggests that cancer stem cells (CSC) may cause tumor recurrence if they are not eradicated by current therapies as radiotherapy or radio-chemotherapy. Recent *in vitro* studies have demonstrated that CSCs may be protected from treatment-induced death by multiple intrinsic and extrinsic mechanisms. Therefore, early determination of CSC abundance in tumor biopsies prior-treatment and development of therapeutics, which specifically target CSCs, are promising strategies to optimize treatment. Here we provide evidence that aldehyde dehydrogenase (ALDH) activity is indicative for radioresistant HNSCC CSCs. Our study suggests that ALDH^+^ cells comprise a population that maintains its tumorigenic properties *in vivo* after irradiation and may provide tumor regrowth after therapy. We found that ALDH activity in HNSCC cells can be attributed, at least in part, to the ALDH1A3 isoform and inhibition of the ALDH1A3 expression by small interfering RNA (siRNA) decreases tumor cell radioresistance. The expression dynamic of ALDH1A3 upon irradiation by either induction or selection of the ALDH1A3 positive population correlates to *in vivo* curability, suggesting that changes in protein expression during radiotherapy are indicative for tumor radioresistance. Our data indicate that ALDH1A3^+^ HNSCC cells may contribute to tumor relapse after irradiation, and inhibition of this cell population might improve therapeutic response to radiotherapy.

## INTRODUCTION

Approximately 600,000 new cases of head and neck squamous cell carcinoma (HNSCC) are diagnosed yearly worldwide [1]. HNSCC includes a heterogeneous group of malignancies that originate from the mucosal surfaces of the upper aerodigestive tract. Although HNSCC is highly curable at early stages, about 60% of HNSCC patients are diagnosed with loco-regionally advanced disease (stage III–IV), which is associated with poor prognosis [2]. Intensified radiotherapy schemes and combination treatment concepts for advanced disease management contribute to a better outcome and organ preservation. Nevertheless, HNSCC remains a disease associated with an unsatisfactory overall 5-year survival rate of approximately 50% for patients with advanced stage disease at the time of diagnosis [1].

Besides the human papilloma virus (HPV) status, which is associated with better outcome and higher radio- and chemosensitivity, current risk stratification and treatment decision parameters for HNSCC rely on the preoperative evaluation of the clinical data including the anatomic site of the primary tumor, tumor size (T), regional lymph node involvement (N) and presence of distant metastasis (M), which are modestly predictive for the individual patient's outcome [2, 3]. Reliable and predictive evaluation of the patient's specific biological characteristics is highly desirable to allow a better individual tailoring of the existing therapeutic options for each patient and prevent unnecessary treatment-associated toxicity. There is persuading evidence that many tumors are maintained by a population of so called cancer stem cells (CSCs), which are responsible for tumor development, dissemination and relapse [4]. Clinical and preclinical evidence suggest that CSC-related tumor parameters such as pre-treatment numbers of CSCs and their inherent radiosensitivity might influence response to radiotherapy and radiochemotherapy in HNSCC [5, 6]. Such CSC-related predictors of the tumor radiation response include the density of CSCs, their repopulation, reoxygenation, and distinct intrinsic mechanisms such as DNA repair capacity during the course of radiotherapy, which may lead to tumor radioresistance and relapse after therapy [7–9]. Due to the high plasticity of the CSC populations, these characteristics might be dynamic, and could change the cell intrinsic and extrinsic therapy response throughout the course of treatment [7].

Several markers have been proposed to identify CSCs in HNSCC, including CD133, CD44, ATP-binding cassette sub-family G member 2 (ABCG2), stemness-related transcription factors Nanog, Octamer binding transcription factor 4 (Oct4), sex determining region Y (SRY)- box 2 (Sox2), and aldehyde dehydrogenase (ALDH) activity [6, 10–14]. In this study, we aimed to investigate the irradiation dependent expression of those CSC markers and their relation to radioresistance. We found that ALDH activity, which is, at least partially, attributed to ALDH1A3 isoform, is indicative of those HNSCC tumor progenitors. Its expression in xenografts correlates with the occurrence of the CSC marker Oct4 and phosphorylated pro-survival Protein kinase B (pAkt). In contrast to ALDH negative cell populations, ALDH positive cells maintain their tumorigenic properties *in vivo* after irradiation, and may contribute to tumor relapse. Our study also suggests that not only the marker expression prior treatment, but rather expression dynamics of ALDH1A3 upon therapy correlates with tumor radiosensitivity.

## RESULTS

### Generation and characterization of radioresistant sublines of HNSCC cells

**Figure 1:**
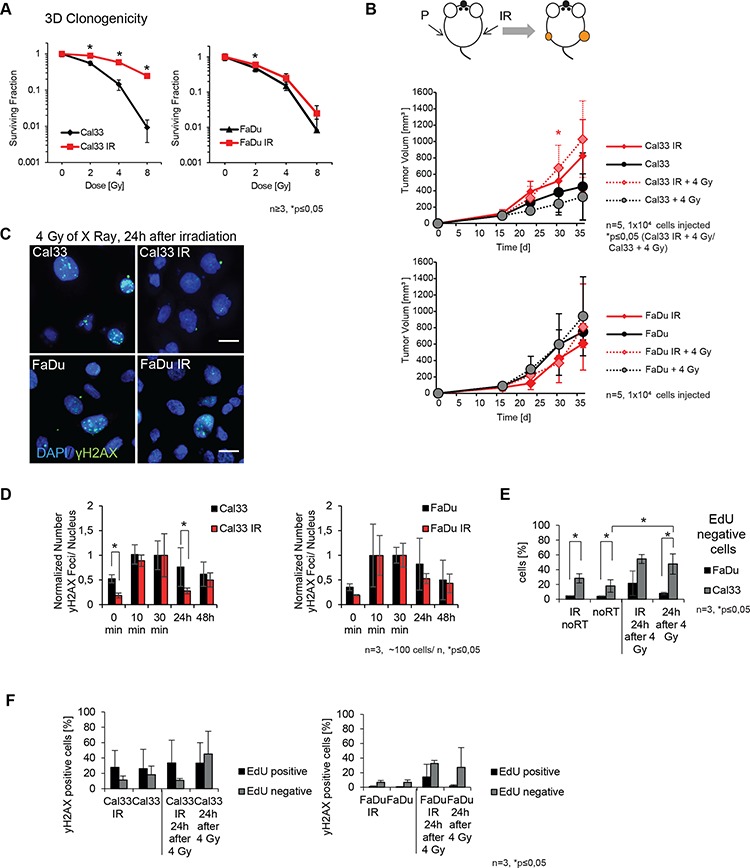
Establishment and characterization of irradiated HNSCC sublines **A.** 3D radiobiological survival colony formation assay comparing irradiated (IR) versus parental FaDu and Cal33 lines. **B.** Tumor volume measured for Cal33 and FaDu and their respective IR sublines. The size of xenograft tumors derived from Cal33 IR cells and measured at day 30 is significantly larger as compared to the tumors formed by the Cal33 parental cells when they were irradiated before injection (*n* = 5). **C.** Immunofluorescence images of γH2AX foci 24 h after irradiation (blue: DAPI, green: γH2AX foci, scale bar is 20 μm). **D.** Normalized mean number of γH2AX foci towards the 30 min value of initial damage at different time points after 4 Gy irradiation for FaDu and Cal33 parental and IR HNSCC lines. **E.** Comparison of distribution of DNA synthesizing cells of Cal33 and FaDu within 24 h with or without irradiation. **F.** γH2AX positive cells within the EdU negative and EdU positive fraction comparing parental and IR sublines of Cal33 and FaDu without irradiation or 24 h after irradiation (*n* = 3 for FaDu and Cal33 for γH2AX assays, *n* > 3 for clonogenic assays, *n* = 5 for tumor growth, *p* < 0.05, error bars = SD).

One of the mayor challenges in radiotherapy is the prediction of the patient's tumor radioresistance in response to irradiation in order to optimize the given dose for a maximal tumor kill and minimal normal tissue damage [15]. As a tool to identify markers for radioresistance of HNSCC, we generated irradiated sublines (IR) of the established HNSCC cell lines FaDu and Cal33. For this, the cell cultures were treated with multiple fractions of 4 Gy of X-rays to a total dose of more than 56 Gy (Figure S1A). This regimen was chosen to mimic hypofractionated radiation therapy for HNSCC patients with locally advanced and metastatic disease [16]. To characterize the newly established IR sublines, we investigated the cell viability and clonogenic survival upon irradiation as well as tumorigenicity *in vivo* in comparison to the isogenic parental cell lines. The radiobiological 2D and 3D clonogenic survival assays revealed a higher radioresistance of the irradiated HNSCC sublines compared to the non-irradiated parental cell lines, with a slight increase in cell survival for FaDu IR that was significant just at 2 Gy in 3D (and at 2 and 4 Gy in 2D). In contrast, Cal33 IR cells showed a significant increase in radioresistance as compared to parental Cal33 cells that was observed at all given doses (Figure 1A, Figure S1B and S1C). To analyze if the irradiated sublines are able to form tumors *in vivo*, they were subcutaneously injected into NMRI nu/nu mice (1 × 10^4^ cells/mouse) and compared to the tumorigenic potential of their parental counterparts, with or without irradiation of 4 Gy directly before injection. FaDu IR and Cal33 IR sublines as well as the non-irradiated parental cells formed tumors in all injected mice (Figure 1B). Whereas the Cal33 IR subline grew faster than its parental line, FaDu IR cells showed a slower tumor growth compared to the parental cells. Both parental as well as the IR sublines show similar viability suggesting that the above described effects are attributed to the cell intrinsic tumorigenic properties (Figure S1D).

The survival of cells after radiation damage depends on the balance between DNA damage formation and damage repair. The number of radiation-induced γH2AX foci was used as a surrogate marker for DNA double strand break repair efficacy and was analyzed in the irradiated versus parental FaDu and Cal33 cells by immune fluorescent staining (Figure 1C). To determine potential differences of parental and IR sublines in DNA repair ability, the number of γH2AX foci was counted before irradiation, and at 10 min, 30 min, 24 h, and 48 h after irradiation with a 4 Gy dose, and was normalized to the number of γH2AX foci 30 min after irradiation as the initial damage value. Noteworthy, the Cal33 IR subline showed significantly less absolute number of basal at 0 min and also residual γH2AX foci at 24 hours after irradiation than its parental line while the parental and FaDu IR did not differ in the number of residual γH2AX foci (Figure 1D). The lower absolute number of basal γ-H2AX foci in Cal33 IR compared to the parental Cal33 is in line with the significantly higher *in vitro* radioresistance of Cal33 IR and its increased tumor volume growth *in vivo* compared to the parental Cal33 cells (Figure 1A and 1B). The DNA content of both parental and IR sublines of Cal33 and FaDu was the same (Figure S1E). These observations suggest that basal changes in DNA damage response in Cal33 may be one of the cell adaptations to irradiation. In contrast, we observed only minor and not significant differences of basal and residual foci numbers in FaDu IR cells compared to the parental FaDu cell line, which is consistent with the only slight differences in radiosensitivity between FaDu and FaDu IR cells as determined by the colony formation assay. To investigate potential changes in the cell cycle between parental and IR sublines, which could affect γH2AX foci formation after irradiation we performed cell cycle analysis by adding 5-ethynyl-2 deoxyuridine (EdU) to the cells directly before irradiation. Foci formation of γH2AX was measured in EdU positive versus EdU negative cells. No significant differences in cell cycle distribution or the proportion of EdU positive or EdU negative cell population was found comparing the parental and IR sublines (Figure 1E and Figure S1G). Interestingly, Cal33 parental and IR cells have a significantly higher proportion of EdU negative cells in comparison to FaDu, although the proliferation speed of both cell lines is not different (Figure 1E and Figure S1H). Additionally we observed a higher percentage of γH2AX positive cells within the EdU positive fraction of Cal33 in contrast to FaDu cells that show the opposite effect with higher damage in the EdU negative fraction (Figure 1F). Although these differences are not significant, they might hint to the mechanisms of higher radioresistance gained by the Cal33 IR line in comparison to FaDu IR. When we analyzed the cell cycle distribution irrespective of EdU uptake during S-phase, we found for both, the parental lines of Cal33 and FaDu, more γH2AX positive cells in the G2/M phase compared to IR sublines (Figure S1F and S1G). This might explain at least in part the lower number of basal γH2AX foci in the un-irradiated Cal33 IR sublines. Taken together, we have established two different models for a slightly (FaDu/ FaDu IR) and substantially increased cell radioresistance (Cal33/ Cal33 IR) upon irradiation for further investigation of the underlying CSC marker expression in response to radiotherapy.

### Dynamics of the stem cell marker expression in HNSCC cells in response to irradiation

**Figure 2:**
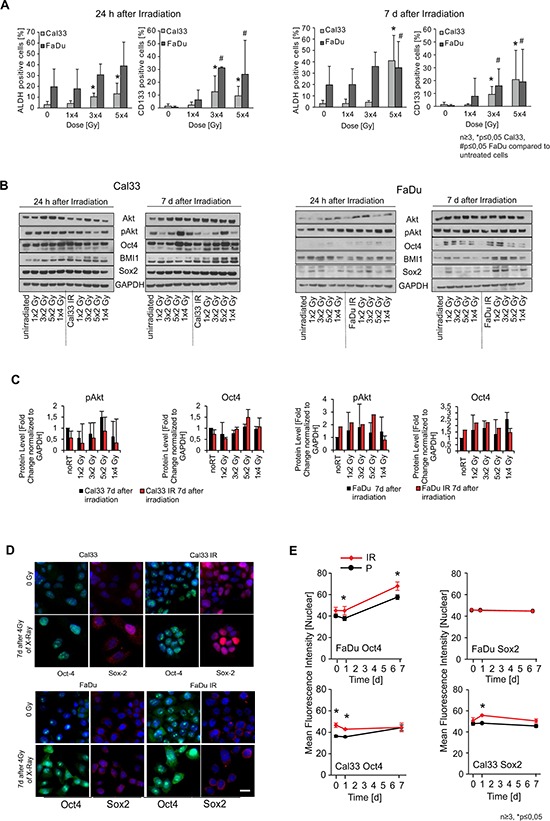
Putative cancer stem cell (CSC) marker expression at different time points after irradiation treatment **A.** Flow cytometric analysis of ALDH activity and CD133 surface expression after 0, 1, 3, 5 times 4 Gy of X-rays, measured 24 h and 7 d after the last irradiation fraction. Significances are depicted for FaDu (#) and Cal33 (*). **B.** Qualitative Western blot analysis of CSC marker expression depending on the number of 2 and 4 Gy fractions and analyzed 24 h and 7 d after last irradiation in parental and IR sublines of Cal33 and FaDu. **C.** Quantitative protein expression levels (fold change of protein expression normalized to GAPDH expression level). **D.** Immunofluorescence analysis of the nuclear localization of the transcription factors Oct4 and Sox2 (blue: DAPI, green: Oct4, red: Sox2, scale bar 20 μm). **E.** Quantification of the mean fluorescence nuclear intensity of Oct4 and Sox2 was performed at 0 Gy, 24 h, and 7 d after 4 Gy of X-rays. (*n* ≥ 3, *p* < 0.05, error bars = SD).

A number of studies conclude that CSC are virtually resistant to radiotherapy and may lead to tumor relapse after therapy [17]. To analyze the radiation-induced changes in the populations of cells with CSC associated phenotypes, we assessed the expression of CSC markers during the generation of the IR sublines. Analysis of the HNSCC cells 24 h and 7 days after X-ray irradiation given as a single dose or fractions of 2 Gy or 4 Gy revealed dynamic changes in the expression of CSC markers. With increasing number of X-ray fractions, the percentage of cells with a high ALDH activity increased as measured by flow cytometry analysis 24 h after X-ray treatment and remained elevated for at least 7 d after the last irradiation (Figure 2A). Analysis of the cell viability revealed no significant changes suggesting that the increase of the ALDH^+^ population rather is induced than caused by selection of the ALDH^+^ cells (Figure S2A). The CSC marker CD133 also increases significantly after three and more fractions of 4 Gy as measured 24 h after irradiation and also remained up-regulated when analyzed 7 d after the last fraction. Similar observations were made for two further HNSCC cell lines, namely UTSCC8 and SAT, where fractionated radiotherapy increased ALDH activity and CD133 expression (Figure S2B). Western blot analysis showed that fractionated irradiation of Cal33 and FaDu cells led to an up-regulation of the expression of the self-renewal and DNA repair related protein BMI1 as well as the stemness-related transcription factors Sox2 and Oct4. We also observed that irradiation induced an up-regulation of the pro-survival Akt phosphorylation (Figure 2B and 2C, Figure S3A and S3B). Similar to the ALDH activity and CD133 cell surface expression, the level of Sox2, Oct4, BMI1, Akt and phospho-Akt remained upregulated at least 7 days after the last irradiation. In addition, Cal33, SAT, and UTSCC8 HNSCC cell lines similarly showed upregulated Oct4 expression 7 d after irradiation (Figure S2C). We also observed those time dependent changes in the expression levels of CSC markers up to five weeks after the last dose (Figure S3C–F). Since the stem cell transcription factors Sox2 and Oct4, which we found to be regulated upon radiation treatment, require nuclear localization for the regulation of gene transcription, we analyzed the nuclear mean immunofluorescence intensity of Sox2 and Oct4 proteins in parental and IR sublines for both FaDu and Cal33 cells upon irradiation, which may be indicative for their involvement in gene expression regulation. The nuclear accumulation of both transcription factors was changed over time after irradiation. As described above for the total protein level, the IR sublines showed a different irradiation-dependent expression dynamic of nuclear Sox2 and Oct4 in comparison to the parental lines (Figure 2D). Nuclear localization of Oct4 was significantly upregulated in unirradiated Cal33 IR cells, whereas FaDu IR sublines showed an upregulation after irradiation (Figure 2E). However, while nuclear localization of Sox2 does not differ between FaDu parental and IR sublines, Cal33 IR sublines showed an elevated level of nuclear Sox2 upon irradiation compared to the parental Cal33 even though we found decreased Sox2 overall expression in Cal33 IR cells as compared to their parental line (Figure S3E and S3F). These changes in transcription factor localization suggest an adaptive response of both Cal33 and FaDu cells after irradiation, which may lead to changes in CSC marker expression and can render the cells more resistant to radiotherapy.

### HNSCC cells positive for ALDH activity are radioresistant

**Figure 3:**
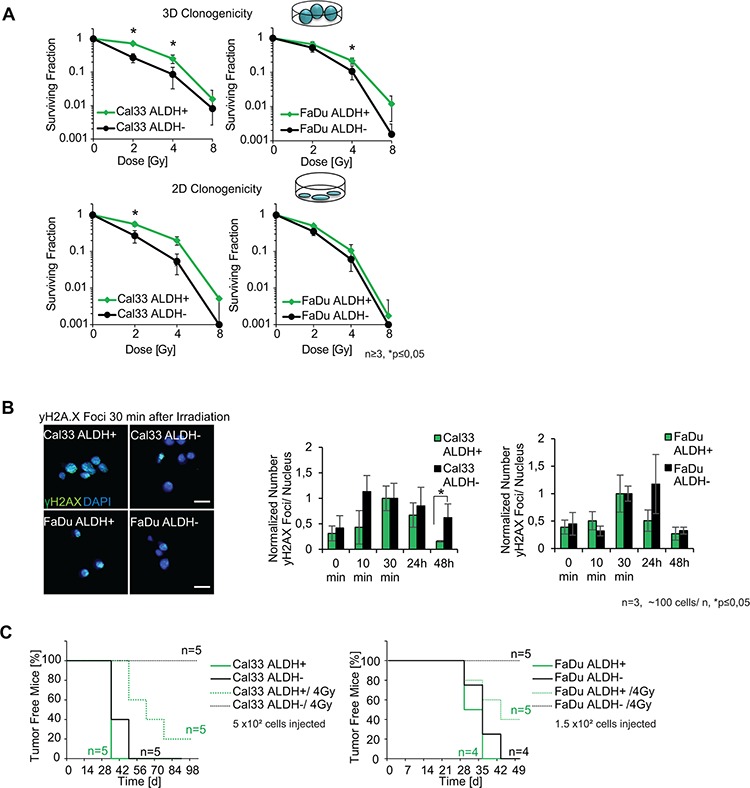
ALDH as a biomarker for radioresistance **A.** Radiobiological clonogenic survival of Cal33 ALDH^+^ and FaDu ALDH^+^ cells as compared to ALDH^−^ populations after irradiation with 2, 4 and 8 Gy of X-ray. **B.** Normalized mean number of γH2AX foci formed after 4 Gy of X-ray irradiation for ALDH^+^ and ALDH^−^ FaDu and Cal33 HNSCC lines at different time points after irradiation. Immunofluorescence images show γH2AX foci 30 min after 4 Gy of X-ray (green: γH2AX, blue: DAPI, scale bar 20 μm). **C.** Tumor free survival after injection of ALDH^+^ or ALDH^−^ HNSCC cells, with (dashed line) or without (solid line) 4 Gy of X-ray irradiation before injection. (*p* < 0.05, error bars = SD).

ALDH has been reported previously as a stem cell marker in HNSCC [11]. However, contradictory roles of ALDH were reported regarding therapy resistance [18, 19]. ALDH catalyzes the oxidation of toxic aldehydes, which are generated e.g. by oxidative stress, and is therefore predestinated to be involved in the regulation of oxidative stress response caused by radiation therapy. As described above, we found that ALDH activity is up-regulated during fractioned irradiation. To test its potential role in the regulation of cellular radioresponse, we employed 2D and 3D radiobiological clonogenic survival assays, as for the IR and parental sublines, to analyze the *in vitro* radioresistance of the cells with a high versus low ALDH activity (ALDH^+^ and ALDH^−^ cell populations, respectively) isolated from Cal33 and FaDu HNSCC cell lines (Figure S4B and S4C). The radiobiological survival assays demonstrated a higher radioresistance of Cal33 ALDH^+^ cells as compared to ALDH^−^ cells (Figure 3A). FaDu ALDH+ cells also tend towards a higher resistance than ALDH^−^ cells but only show a significant increase in the radiation resistance in the 3D survival assay after irradiation with 4 Gy (Figure 3A). Along with this observation, we found that ALDH^+^ Cal33 cells had a fewer number of residual γ-H2AX foci 48 hours after irradiation than ALDH^−^ cells suggesting a more efficient DSB repair in ALDH^+^ cell subset of Cal33 (Figure 3B, Figure S4A). The FaDu ALDH^+^ cells did not show any significant differences in the numbers of basal or residual γ-H2A.X foci as compared to FaDu ALDH^−^ cells (Figure 3B, Figure S4C).

Next, we compared the relative tumorigenicity of the ALDH^+^ and ALDH^−^ populations of FaDu and Cal33 cells in the subcutaneous nu/ nu NMRI mice xenograft model with or without a 4 Gy irradiation of the cells before injection (Figure 3C). Interestingly, when the cells were irradiated before s. c. injection, the ALDH^−^ population from both Cal33 and FaDu lost its tumorigenic potential and did not form tumors in contrast to ALDH^+^. These results suggest that unlike ALDH^−^ cells, the ALDH^+^ cell population maintains its tumorigenic potential after irradiation (Figure 3C, Figure S5A). Interestingly, when we isolated ALDH^+/−^ cell populations from IR cell lines and performed the tumorigenic *in vivo* assay in the nu/ nu NMRI mouse model, we discovered that also the ALDH^−^ population was able to form tumors after irradiation (Figure S5B). We excluded the possibility that this effect was due to different viability of the sorted cells (Figure S5C). This suggests that in contrast to non-irradiated tumor cells, IR tumor cells do not depend on the ALDH activity anymore as a potential radiation-defense mechanism.

### ALDH-dependent radioresistance of HNSCC cells is attributed to ALDH1A3 isoform

**Figure 4:**
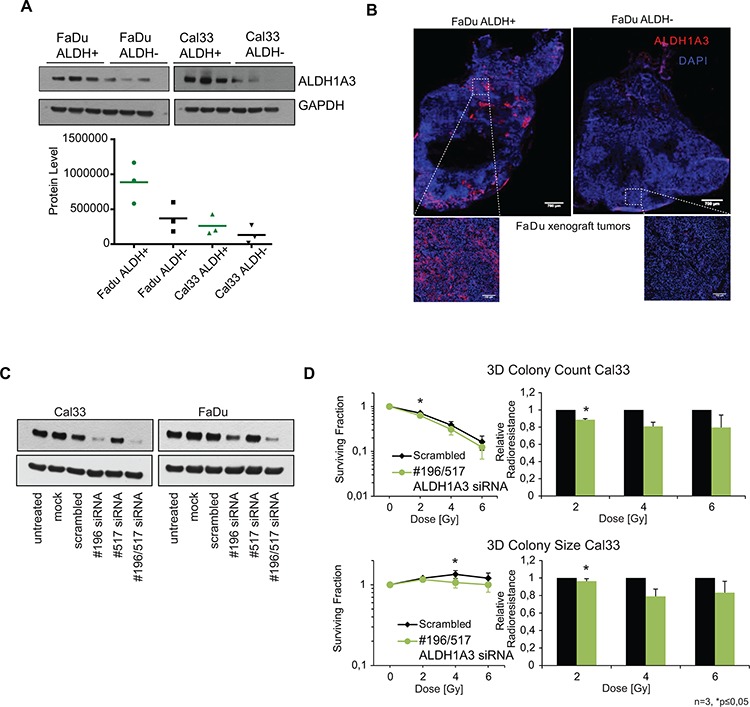
ALDH1A3 as radioresistance relevant enzyme **A.** Qualitative and quantitative western blot analysis of ALDH1A3 expression in xenograft tumors derived from ALDH^+^ or ALDH^−^ FaDu and Cal33 cells. **B.** Immunofluorescence analysis of the ALDH1A3 expression in FaDu xenografts derived from ALDH^+^ and ALDH^−^ cells (blue: DAPI, red: ALDH1A3, scale bar: overview whole tumor 700 μm, scale bar for zoomed in image 100 μm). **C.** Western blot analysis of the siRNA knockdown efficacy of ALDH1A3 in FaDu and Cal33. **D.** Reduction of the ALDH1A3 expression by siRNA results in an increase in cell radiosensitivity of Cal33 cells as compared to the siRNA control cells. (*n* = 3, *p* < 0.05, error bars = SD).

The isolation of ALDH^+^ and ALDH^−^ populations for the clonogenic and tumorigenic survival assays was based on the Aldefluor®assay. It was demonstrated before that ALDH activity measured by the Aldefluor®assay is attributed to ALDH1 and ALDH2 isozymes [20]. Recent data also showed that ALDH activity might be associated with expression of ALDH isozymes 1A1 and 1A3 [21, 22]. Analysis of the mRNA expression level of those ALDH isoforms in parental and IR sublines of FaDu and Cal33 cells revealed that FaDu cells have a very low *ALDH1A1* expression, and both, Cal33 and FaDu cells are positive for the expression of *ALDH1A3* (Figure S6A). These results were also confirmed by the immunofluorescent analysis of ALDH1A3 and ALDH1A1 expression in HNSCC cells (Figure S6B). In agreement with the q-PCR results, the ALDH1A3 protein level was increased in IR FaDu sublines in response to irradiation (Figure S6C). Western blot analysis of the ALDH1A3 protein level in xenograft tumors derived from ALDH^+^ and ALDH^−^ cells of both tumor models, Cal33 and FaDu, revealed high ALDH1A3 protein level in the xenografts grown from ALDH^+^ cells, which kept their tumorigenicity *in vivo*, in contrast to the tumors established from ALDH^−^ cells, which lost their tumorigenic potential upon radiation (Figure 4A). These results were confirmed by immunofluorescence staining of the xenograft tumors derived from FaDu ALDH^+^ or FaDu ALDH^−^ cells (Figure 4B). To analyze the impact of ALDH1A3 expression on the cell radiosensitivity, we performed siRNA knockdown of the ALDH1A3 expression in both FaDu and Cal33 cells. Reduction in the ALDH1A3 protein expression was confirmed by Western blotting (Figure 4C). We employed 3D radiobiological clonogenic assays to measure the relative radioresistance of cells with a high versus low ALDH1A3 expression. Reduction of ALDH1A3 expression by targeting two sequence positions on ALDH1A3 mRNA resulted in a slight, but significant increase in cell radiosensitivity of Cal33 cells as compared to the siRNA control cells (Figure 4D). Remarkably, not only the number, but also the size of the 3D colonies formed by the Cal33 cells transfected with ALDH1A3 siRNAs was decreased after irradiation (Figure 4D). Despite the effect of ALDH1A3 knock down on the Cal33 cell radioresistance and colony size was significant, this effect was less pronounced in FaDu cells. This might be due to a high level of endogenous ALDH1A3 expression in FaDu cells, impeding an efficient inhibition of ALDH1A3 expression (Figure 4C). The observation might also indicate that, in addition to ALDH1A3, some other ALDH isozymes are involved in the ALDH activity of FaDu cells, which might compensate for the ALDH1A3 knockdown. Analysis of gene expression of the ALDH isozymes demonstrated that the FaDu IR subline additionally expresses *ALDH3A1* that could potentially be involved in the FaDu ALDH^+^ cell population in contrast to Cal33 IR, which shows downregulation of that ALDH variant (Figure S6D). Taken together, these results suggest that ALDH-dependent cellular radioresistance may be, at least in part, attributed to the ALDH1A3 isozyme.

### Correlation of ALDH1A3 expression with expression of Akt, Oct4, and CD44 as established HNSCC biomarkers *in vivo*

**Figure 5:**
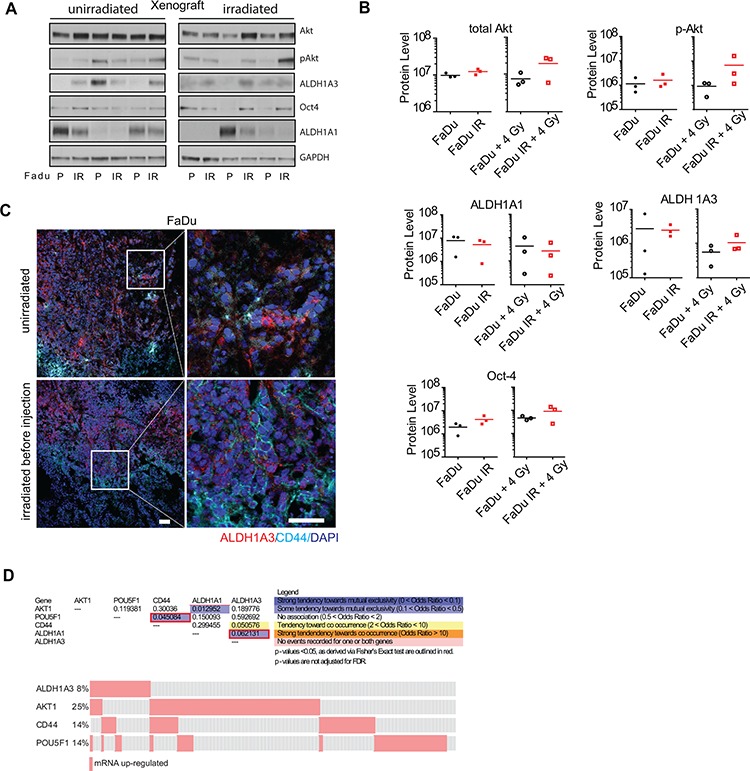
Expression of ALDH1A3 protein in xenograft tumors and human primary HNSCC tissues **A.** Western blot analysis of the xenograft tumors formed by the parental and IR FaDu cells. **B.** Level of the protein expression analyzed by western blotting and normalized to the level of the GAPDH expression. Each dot represents one xenograft. **C.** Staining of CD44 and ALDH1A3 in xenograft tumors formed by FaDu cells (scale bar 50 μm) **D.** Comparative mRNA expression analysis of ALDH1A3 in human data set; upper panel: calculated co-occurrence of *ALDH1A3, CD44, AKT1* and *Oct4* using TCGA data set of 517 non-randomized tumors. The results shown here are in whole based on the data from the TCGA Research Network: http://cancergenome.nih.gov/. lower panel: oncoprint of the HNSCC cases with upregulated expression of genes *ALDH1A3, CD44, AKT1* and *Oct4(POU5F1)*.

To investigate whether our findings *in vitro* correspond to *in vivo* conditions, we analyzed the protein expression levels of ALDH1A3, total Akt, phospho-Akt, and Oct4 in xenograft tumors formed by FaDu parental and IR cells, which were irradiated or remained non-irradiated before injection (Figure 5A). Quantification of the protein levels revealed an increase of Akt, phospho-Akt, Oct4, and ALDH1A3 in the xenograft tumors that originated from the pre-irradiated FaDu IR cells as compared to the xenografts grown from the pre-irradiated parental cells (Figure 5B). Interestingly, even though we were not able to detect ALDH1A1 *in vitro*, we found ALDH1A1 expression in xenograft tumors, where its expression inversely correlates with the expression of ALDH1A3, Akt, phospho-Akt, and Oct4 (Figure 5A and 5B). Immunofluorescent analysis of these xenograft tumors showed a co-staining of ALDH1A3 and CD44, which is a previously described CSC marker for HNSCC [19] (Figure 5C). In line with these results, cBioPortal analysis of a TCGA HNSCC data set for 517 tumor specimens showed that expression of *ALDH1A3* had a tendency toward co-occurrence with *CD44* expression, whereas expression of *ALDH1A1* and *AKT1* as well as *ALDH1A1* and *ALDH1A3* genes tended to be mutually exclusive (Figure 5D).

### Correlation of ALDH1A3 expression with tumor stage and radiotherapy response

**Figure 6:**
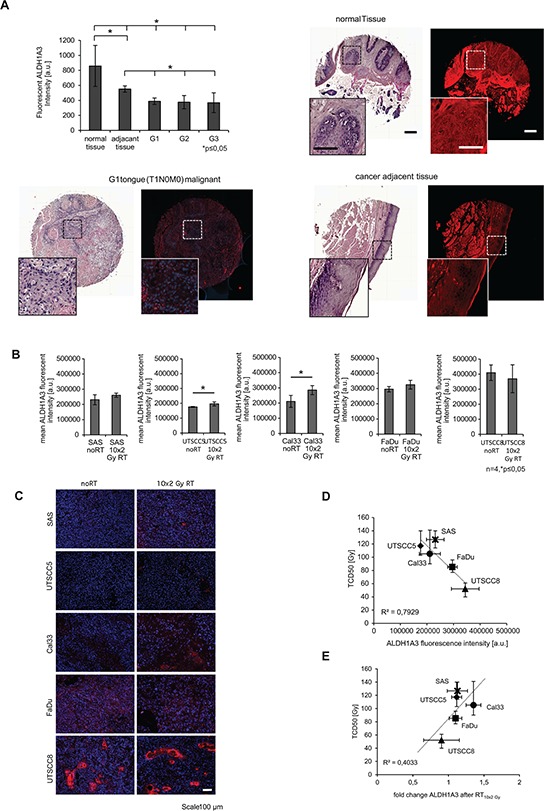
Correlation of ALDH1A3 expression to tumor radiosensitivity **A.** Staining of ALDH1A3 in human primary tumors with different grades (scale bar whole tumor core 200 μm, scale bar zoom in 100 μm). **B.** mean immunofluorescent intensity of ALDH1A3 expression without and with radiotherapy (RT) of 10 × 2 Gy. **C.** Immunofluorescent images of the five investigated xenografts (scale bar 100 μm). **D.** Correlation of ALDH expression intensity before radiotherapy. **E.** Correlation of expression level changes of ALDH1A3 fluorescent intensity to the TCD50 of the xenograft tumors. (*n* ≥ 3, *p* < 0.05, error bars = SD, TCD50 errors bars = CI 95%).

To investigate whether ALDH1A3 correlates with tumor stages and bears potential as a predictor of the response to radiotherapy, we investigated its expression in a human HNSCC tumor microarray (TMA) and correlated immunofluorescence intensity with tumor stage. Surprisingly normal tissue and cancer adjacent tissue showed significantly higher ALDH1A3 intensity compared to the tumor tissues. In addition, we did not detect differences in ALDH1A3 expression between the different tumor stages (Figure 6A). Therefore we evaluated the ALDH1A3 fluorescent intensity in xenografts, which were grown from five different HNSCC lines that vary in their radiocurability, namely SAS, UTSCC5, Cal33, FaDu, and UTSCC8, [5]. We determined the ALDH1A3 mean fluorescent intensity of non-irradiated tumors versus 10 × 2 Gy irradiated tumors that were dissected 24 h after the last dose (Figure 6B and 6C). We found a negative correlation of ALDH1A3 expression and the tumor control dose 50 (TCD50), which reflects the overall radiosensitivity of each tumor model (Figure 6D). This goes along with the results obtained for the human TMA. However, comparison of the xenografts that were treated 10 × 2 Gy fractions with untreated xenografts of the same HNSCC line revealed changes of ALDH1A3 fluorescence intensity that positively correlated with the TCD50 values of the five tumor models (Figure 6E). For Cal33 and UTSCC5 we found a significant increase of ALDH1A3 fluorescence intensity after radiotherapy (Figure 6B). Taken together, high ALDH1A3 intensity was found in non-tumorous, cancer adjacent human tissues as well as in radiosensitive tumor models, whereas radioresistant tumor models exhibited a low ALDH1A3 expression prior therapy which then increased upon treatment.

## DISCUSSION

Significant progress in understanding the molecular mechanisms of tumor response to irradiation enabled the development of a number of potential biomarkers for radiotherapy individualization in HNSCC, including epidermal growth factor receptor (*EGFR*) gene expression, DNA repair efficacy of cancer cells, tumor hypoxia and glucose uptake, human papillomavirus (HPV) status, and expression of CSC markers [7, 8, 23]. During the last decade, a large number of CSC markers was identified that are potentially involved in therapy resistance and tumor relapse [4, 7, 8, 23]. Among these CSC-specific antigens, a high expression of surface markers CD133, CD44, ABC-family transporter proteins such as ABCG2, transcription regulators Nanog, Oct4, Sox2 and ALDH activity were assigned for potential CSC populations in HNSCC [6, 10–12, 24, 25]. A few recent retrospective clinical studies correlated CSC phenotypes to radio-chemotherapy outcome in HNSCC including expression of CD44, CD24, integrin β1 and Oct4 [14, 18, 19]. These studies suggested that the quantification of CSC numbers in pre-treatment tumor biopsies could be a useful prognostic marker to identify those patients, which may benefit from radiotherapy or from combination of radiation and CSC-targeted therapy.

In this study we aimed to investigate the potential role of CSC marker expression in HNSCC radiosensitivity and their regulation upon radiotherapy. We showed an up-regulation of stem cell markers in HNSCC cells after irradiation including expression of CD133, ALDH activity, as well as expression of the proteins involved in the regulation of survival, stemness and self-renewal, such as Akt, Oct4, and BMI1. Irradiation of FaDu and Cal33 cells with multiple 4 Gy fractions led to an acquisition of long term moderate radioresistance in FaDu cells and substantially increased radioresistance in Cal33 cells compared to their parental lines. As shown before by Yaromina et al. parental Cal33 cells exhibit high radioresistance. Nevertheless the IR derivatives of Cal33 are even more radioresistant. In contrast parental FaDu cells are only moderately resistant to radiotherapy and their IR daughters only slightly increased their radioresistant properties [5]. This acquired cellular radioresistance might be attributed to the selection of the pre-existing minor cellular clones or to de-novo mutations or epigenetic changes that occur in the cells during or after irradiation. Indeed, recent investigations demonstrated that cancer therapy represents a strong selection pressure for tumor cells and that high intra-tumor genetic diversity correlates with only partial or no therapy response and worse treatment outcome in HNSCC cancer patients [26, 27]. This suggests that high heterogeneity within one tumor may be attributed to various mutations within tumor progenitor cells that could favor their survival during cancer therapy and increase the possibility of tumor regrowth. Other studies also reported that anti-cancer therapy can effect clonal evolution and lead to the emergence of minor mutated clones, which are therapy resistant, and associated with tumor relapse [28]. Moreover, clonal expansion of tumor cells with advantageous mutations leading to therapy resistance and poor survival has been demonstrated to be clinically relevant in different types of cancer [29, 30]. In addition to the treatment-related clonal selection, recent evidence suggests that radiotherapy can directly sustain cancer cell de-differentiation to a stem cell phenotype that can potentially impact tumor curability [31]. However, the question whether the emergence of radioresistant HNSCC sublines is an induced or selective response to irradiation requires further investigation. Future studies utilizing *in vivo* tracking of CSCs will clarify the effect of irradiation on the induction and selection of radioresistant clones.

The high radioresistance of the Cal33 IR subline was associated with reduced numbers of basal as well as residual γH2AX foci as compared to Cal33 parental cells. In contrast the moderate radioresistant FaDu IR model did not exhibit significant changes in DNA damage response.

Our data demonstrate that HNSCC cells with a high ALDH activity were more radioresistant than ALDH^−^ cells. Despite both cell populations, ALDH^−^ and ALDH^+^ are tumorigenic, only the ALDH^+^ cells maintained their tumorigenic properties *in vivo* after pre-irradiation. This is in line with recently published data from Bertrand et al., who showed that inhibition of ALDH activity by the treatment with all-trans retinoid acid (ATRA) decreased the survival of HNSCC cells after photon and carbon irradiation [32]. The ALDH family of proteins are enzymes that are involved in the oxidization of intracellular aldehydes to carboxylic acids and contribute to the synthesis of retinoid acid, which plays a role in the maintenance and differentiation of normal and cancer stem cells. The enzymatic and non-enzymatic functions of ALDH1-family members play a key role in the cellular response to oxidative stress by direct scavenging of radiation-induced free radicals or by producing the antioxidant NAD(P) H [25, 33]. Targeting and/ or inhibition of ALDH1 was found to reduce sphere formation, tumor growth and metastasis [34, 35]. We found that ALDH activity in HNSCC cells can be attributed, at least in part, to the ALDH1A3 isoform of the ALDH protein family and knockdown of ALDH1A3 expression led to increased radiosensitivity of HNSCC cells. Interestingly, the knockdown of ALDH1A3 expression was only significantly efficient in Cal33 of which we were able to establish highly radioresistant IR sublines in contrast to the moderate radioresistant FaDu cell line. But contrary to this, Cal33 cells had much lower ALDH1A3 expression as compared to FaDu cells as we showed by flow cytometry, western blotting, RT-PCR, and immunofluorescence. Since we found only ALDH^+^ cells to be tumorigenic *in vivo* after irradiation, we assumed that a high ALDH1A3 expression rate could correlate with the TCD50 of treated xenografts as well as with the stage of human HNSCC tumors but discovered a negative relation. Therefore, our data suggest that initial ALDH1A3 expression cannot be used to predict the radioresistant potential of tumor cells. Contradictory effects of ALDH1A3 expression on breast cancer progression or suppression were also recently reported by Marcato et al. [36]. They found differential expression of ALDH1A3/ RA inducible genes that promote or suppress tumor growth in dependency of the DNA methylation state. This supports the role of epigenetic regulatory mechanisms in the radioresponse of cells [37]. Also for Non-small-cell lung carcinoma (NSCLC) ALDH1A3 was described as pre-dominant ALDH isoform, which on one hand associated with well differentiated lung cancer with good prognosis but on the other hand drives the tumorigenic and clonogenic potential of NSCLC cells [38]. In the same study they found the Janus kinase/ Signal Transducer and Activator of Transcription (JAK2/STAT3) pathway to contribute to ALDH1A3 expression. The methylation state of the *STAT3* gene was found to be mediated by Enhancer of zeste homolog 2 (EZH2), that participates in DNA methylation and which can be activated by Akt signaling [39]. In line with this, we observed, similar to ALDH activity, an increase in the expression of other investigated CSC markers, e.g. pAkt upon irradiation *in vitro* and *in vivo*. We also observed that the change of the ALDH1A3 expression level before and after radiotherapy may correlate with the tumor cell intrinsic radiosensitivity that could be attributed to an increase in other CSC markers. Supporting this, Raha et al. also found that the ALDH^+^ population shares common properties with drug tolerant CSCs [33]. They discuss, that ALDH activity might be one mechanism to protect the CSCs from toxic side effects of therapy and reactive oxygen species (ROS) [33]. As already stated by Bragado et al. tumors have highly dynamic adaptation mechanisms to stressful stimuli such as therapeutic intervention [13]. This is supported by our findings that ALDH activity is not indicative anymore for radioresistance and tumorigenicity in IR cell populations, since also ALDH^−^ cells derived from IR sublines were able to form xenografts after irradiation. This underlines the importance to search for potential biomarkers not only in untreated cells or tumor biopsies, but also to investigate tumor cells during or after therapy to potentially correlate the dynamics of the marker expression level to potential radioresistance.

In addition, the use of other animal models for the measurement of surviving tumorigenic cells, based on *in vivo* tumor irradiation and combined with a gene reporter - based tracking of the tumorigenic cell populations induced or selected during tumor treatment, might help to elucidate novel biomarkers and regulators of tumor adaptation and evasion upon anti-cancer therapy.

In summary, we established different radioresistant models of HNSCC for *in vitro* and *in vivo* investigation of the traits of radioresistance. Our results suggest that ALDH activity partially mediated by the ALDH1A3 isoform in HNSCC cells may be correlated with radiotherapy outcome depending on its activation level during therapy.

## MATERIALS AND METHODS

### Cell lines and culture conditions

The HNSCC cell lines FaDu (ATCC, Manassas, VA) and Cal33 (DSMZ, Braunschweig, GER) were grown in Dulbecco's Modified Eagle's Medium (DMEM, Sigma-Aldrich) supplemented with 2% HEPES (1 M, PAA Laboratories), 1% Sodium Pyruvate (100 mM, Sigma), 1% MEM non-essential amino acids (100x, Sigma), 10% fetal bovine serum (FBS, PAA Laboratories), and 1% L-glutamine (200 mM, Sigma-Aldrich) according to the manufacturer's recommendations in a humidified 37°C incubator supplemented with 5% CO_2_. All cell lines were genotyped using microsatellite polymorphism analysis. Irradiated (IR) sublines from FaDu and Cal33 were generated via repetitive irradiation of the cell culture with at least 14 fractions of 4 Gy (200 kV X-rays, 0.5 mm Cu filter, 1 Gy/min, Yxlon Y.TU 320). Cells were not passaged during the fractionated irradiation and kept at confluence levels of around 50%. For the 2D and 3D colony formation assays, cells were plated in single cell suspensions. The results of the assays were calculated as plating efficacy: $PE=\frac{\text{counted colonies}}{\text{seeded cells}}\cdot 100$ and surviving fraction: $SF=\frac{\text{counted colonies}}{\text{seeded cells ⋅PE}}\cdot 100$. The siRNA-mediated gene expression knockdown was performed using Lipofectamine® RNAiMAX reagent (Invitrogen) and OptiMEM (Invitrogen) according to manufacturer's protocol.

### Mice and *in vivo* tumorigenicity assays

HNSCC cells were embedded in 100 μl of DMEM/ Matrigel mixed as 1:1 and injected into the flanks of 8 to 12-weeks-old female NMRI (nu/nu) mice (Experimental Centre, Medical Faculty, Technical University Dresden). The animal facility and the experiments were performed according to the institutional guidelines and the German animal welfare regulations (protocol number 24-9168.11-1/2010-21). For immunosuppression, the mice underwent total body irradiation of 4 Gy (200 kV X-rays, 0.5 mm Cu filter, 1 Gy/min) 1 day before injections. Tumor volumes were measured once per week with a digital caliper. The relative tumor volume (mm^3^) was calculated as (length × width × height)/2. For calculation of the tumor free survival, the tumor uptake threshold was set to 100 mm^3^. The mice were observed until a maximum tumor diameter of 15 mm. Xenograft tumors were excised, fixed overnight in 4% para-formaldehyde (Thermo Scientific), and kept 48 h in 30% sucrose before embedding in TissueTek O.C.T compound (Sakura Finetek) and freezing at −80°C. The frozen tumors were cut into 10 μm sections with the Microm HM 560 (Cryo-Star Cryostat). Radiotherapy treated xenografts were established as described in [40] and excised 24 h after the last dose of treatment, snap frozen, and fixed 10 min in ice cold aceton (Roth). Staining procedure was done as described above. For TCD50 correlation, established values were taken from Yaromina et al. [5].

### Immunofluorescent microscopy, flow cytometry and western blotting

Western blot analysis was performed using the cells at the different timepoints after irradiation including 10 min, 24 h, 7 d, 14 d or 5 weeks. The antibodies anti-ALDH 1A1 (Santa Cruz Biotechnology), anti-ALDH 1A3 (Sigma-Aldrich), anti-Akt (Cell Signaling), anti-pAkt (Ser473) (Cell Signaling), anti-BMI1 (Cell Signaling), anti-GAPDH (Cell Signaling), anti-Oct4 (Millipore), and Anti-Sox2 (Santa Cruz Biotechnology) were used according to manufacturer's instruction. For immunofluorescence analysis, the cells were fixed for 10 min in 4% formaldehyde in PBS at room temperature. The cells were washed with PBS, and blocked and permeabilized by incubation with 5% BSA and 0,1% Triton-X100 in PBS. The cells were then incubated with primary antibody diluted in 1% BSA and 0,1% Triton-X100 in PBS overnight at 4°C, and then washed 3 times with PBS. Cells were then incubated for 1 h with a secondary anti-mouse Alexa 488 (Life Technologies), anti-rabbit Alexa 546 (Life Technologies), and DAPI (Sigma). For flow cytometry, the Aldefluor® Kit (Stem Cell Technologies) was used to determine the ALDH activity, and CD133/2-PE (293C, Miltenyi) antibody was used to determine the surface expression of CD133 according to manufacturer's instruction. Cell Cycle measurments were done using the EdU Base Click system (Sigma) according to the manufacturer's instructions. Briefly, the cells were fed with 10 μM EdU and immediately irradiated and left for 30 min, 24 h, or 48 h before fixation. The timepoints were chosen as for the microscopic analysis of γH2AX. After trypsinization the cells were fixed with 2% formaldehyde for 10 min, washed, and fixed another 30 min with methanol on ice. After another washing step, the cells were preceded for γH2AX and Propidium iodide PI (Sigma) staining. For the γH2AX, Oct4, and Sox2 microscopic assays, cells were plated on chamber slides (Millipore), fixed with 4% formaldehyde (Thermo Scientific) for 10 min and stained with anti-γH2AX (Millipore), anti-Oct4 (Millipore), Sox2 (Santa Cruz Biotechnology), and DAPI. The number of γH2AX foci and the Oct4, and Sox2 level were evaluated for at least 100 cells per each experimental condition. A minimum of four random images per repeat were taken and counted. To directly compare differences between time-dependent foci formation between the parental and irradiated sublines, the number of *γ*H2A.X foci in the irradiated cells was normalized to the initial damage foci number at 30 min after irradiation. For nuclear transcription factor level analysis, all images were taken at the same illumination intensity (Axioscope 2 plus, AxioCam MR3, Zeiss) and were processed with the freeware program ImageJ (http://imagej.nih.gov/ij/). Xenograft sections were stained with anti-ALDH 1A3 (Sigma-Aldrich) followed by secondary anti-rabbit Alexa 546 (Life Technologies), anti-CD44-APC (Miltenyi), and DAPI. The human HNSCC paraffin embedded tissue array was purchased from http://Biomax.us (#HN241a) and were supplied with information about TNM, clinical stage and pathology grade. The tissue slides were deparaffinized and rehydrated using standard alcohol series, and the staining procedure was performed as for the xenograft tumor sections. Pictures for immunfluorescence analysis were all taken at the same exposure times and lamp intensities and were evaluated with ImageJ.

### ALDH knockdown, RT-PCR analysis and gene expression of ALDH variants

To inhibit ALDH 1A3 expression two different siRNAs (5′→3′: ALDH 1A3 #196: UAUCUUGGUGAACUUGACCtc and ALDH 1A3 #517: GAGGGUUCUAAUACAGCCCtc, Eurofins) were used. Cells transfected with unspecific siRNA (scrambled siRNA AGGUAGUGUAAUCGCCUU, Eurofins) were used as negative control. The relative radioresistance was calculated by normalization of the ALDH1A3-siRNA treated cells to cells transfected with unspecific scrambled siRNA. RNA isolation was performed using the RNeasy Mini Kit (Qiagen) including on-column DNAse (Qiagen) digestion. The cDNA was prepared using the Superscript II Reverse Transcriptase (Invitrogen). The RT-PCR was run on the StepOnePlus Cycler (Applied Biosciences) using GoTaq Mastermix (Promega) supplemented with CXC reference dye (Promega). Primers were used as following: ALDH1A1 (forward: TCT CGA CAA AGC CCT GAA GT, reverse: TAT TCG GCC AAA GCG TAT TC), ALDH1A3 (forward: CCC TGG AGA CGA TGG ATA CAG, reverse: TCT GAG GGT TCT AAT ACA GCC C), and GAPDH (forward: ACC CAG AAG ACT GTG GAT GG, reverse: AGG TCC ACC ACT GAC ACG TT). The relative mRNA expression was normalized to GAPDH. Gene expression analysis was performed using SurePrint G3 Human Gene Expression 8 × 60K v2 Microarray Kit (Design ID 039494, Agilent Technologies).

### Statistics

The results of the radiobiological colony survival assays, microscopic image analysis, flow cytometry analysis, and tumorigenicity were analyzed by paired *t*-test. All error bars are displayed as standard deviation, the TCD50 errors are displayed as a confidence interval of 95%. Results were considered statistically significant with a *p* – value below 0, 05.

## SUPPLEMENTARY FIGURES


