# Supplementary material for: Cancer stem cell related markers of radioresistance in head and neck squamous cell carcinoma
Source: Oncotarget. 2015 Oct 7;6(33):34494–509. doi: 10.18632/oncotarget.5417 (PMC4741468; doi:10.18632/oncotarget.5417)
Supplement: Supplementary file 1 [file oncotarget-06-34494-s001.pdf]

SUPPLEMENTARY FIGURES

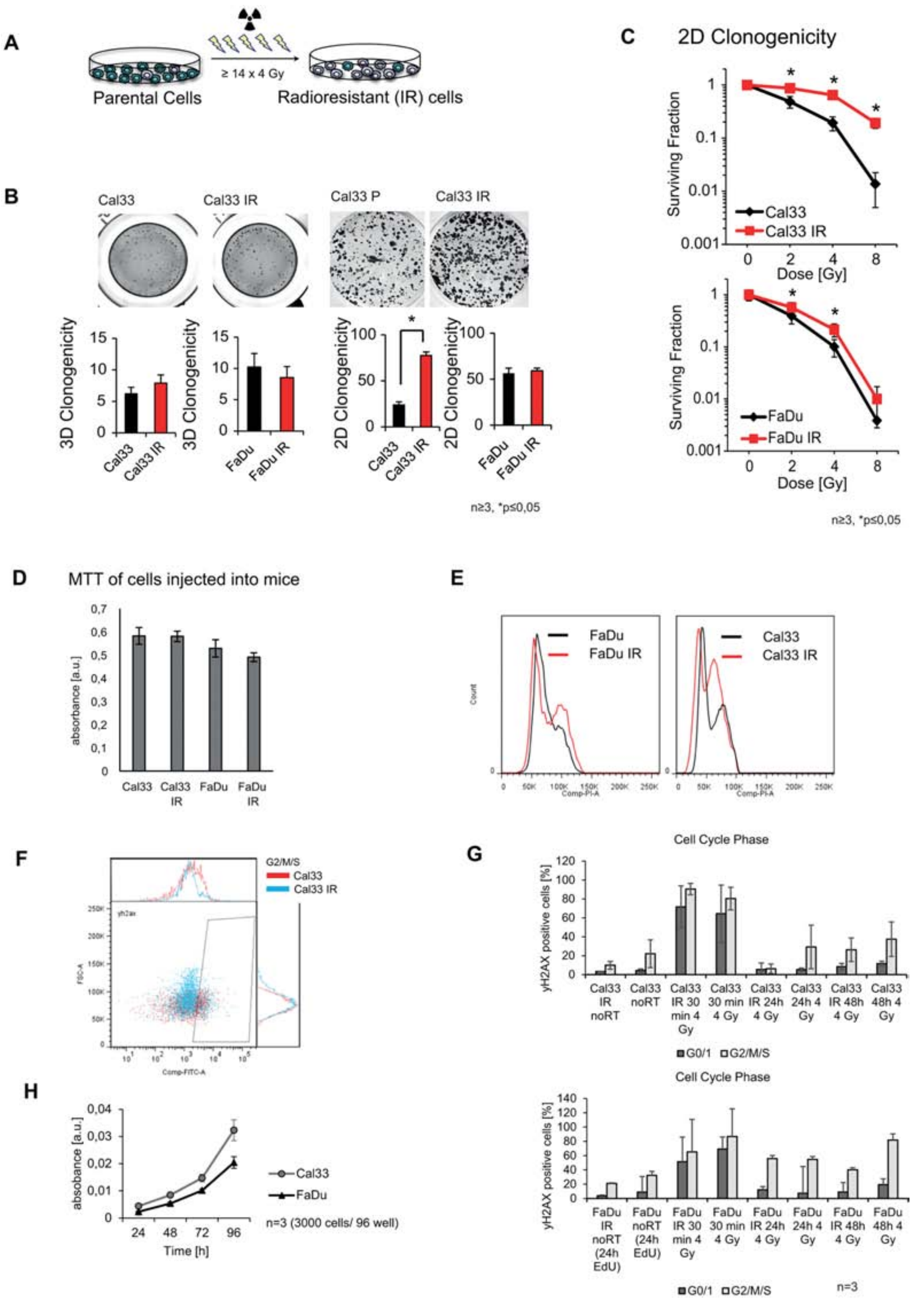

Supplementary Figure S1: Establishment and characterization of irradiated HNSCC sublines. A. IR sublines were established by repetitive irradiation of minimum 14 fractions of 4 Gy. **B.** Clonogenicity of FaDu and Cal33 parental versus IR sublines. **C.** 2D radiobiological survival assay of Cal33 and FaDu parental versus IR sublines. **D.** Viability of the cells injected into mice measured by MTT assay. **E.** DNA content measurement comparing parental and IR sublines of FaDu and Cal33. **F.** flow cytometric dot blot comparing γH2AX foci positive cells of parental and IR Cal33 sublines in the G2/M pahse of the cell cycle. **G.** distribution of γH2AX foci positive cells in the cell cycle phases at different time points after irradiation. **H.** proliferation of Cal33 and FaDu cells measured at different time points after plating 3000 cell/ 96-well. ($n \geq 3$, $p < 0{,}05$, error bars = SD)

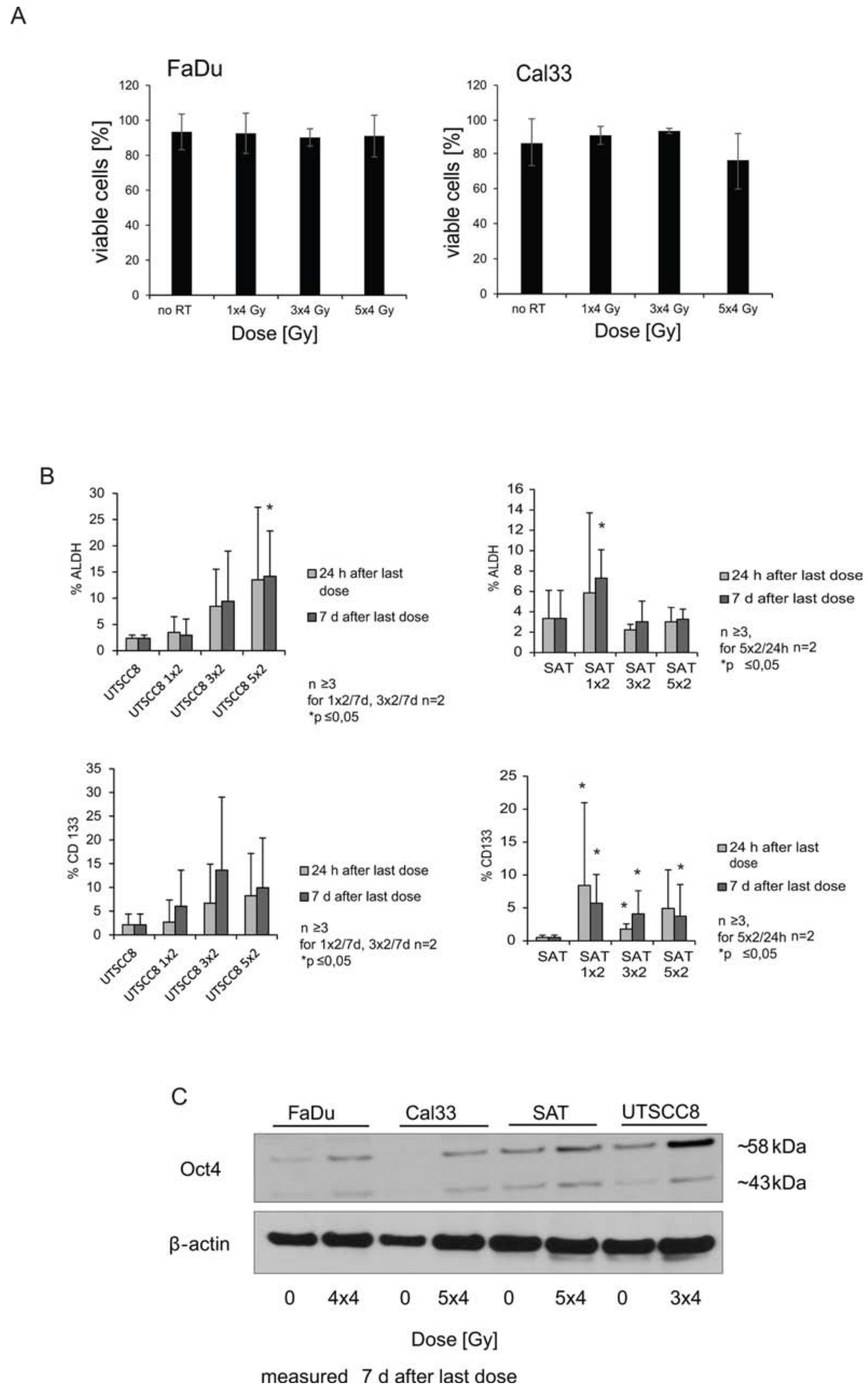

Supplementary Figure S2: Putative cancer stem cell (CSC) marker expression at different time points after irradiation treatment. A. flow cytometric analysis of the cell viability 24 h after the last irradiation fraction using DAPI staining. **B.** flow cytometric analysis of ALDH activity and CD133 surface expression after 0, 1, 3, 5 times 2 Gy of X-rays, measured 24 h and 7 d after the last irradiation fraction. **C.** Western blot analysis of Oct4 expression measured 7 d after the last dose.

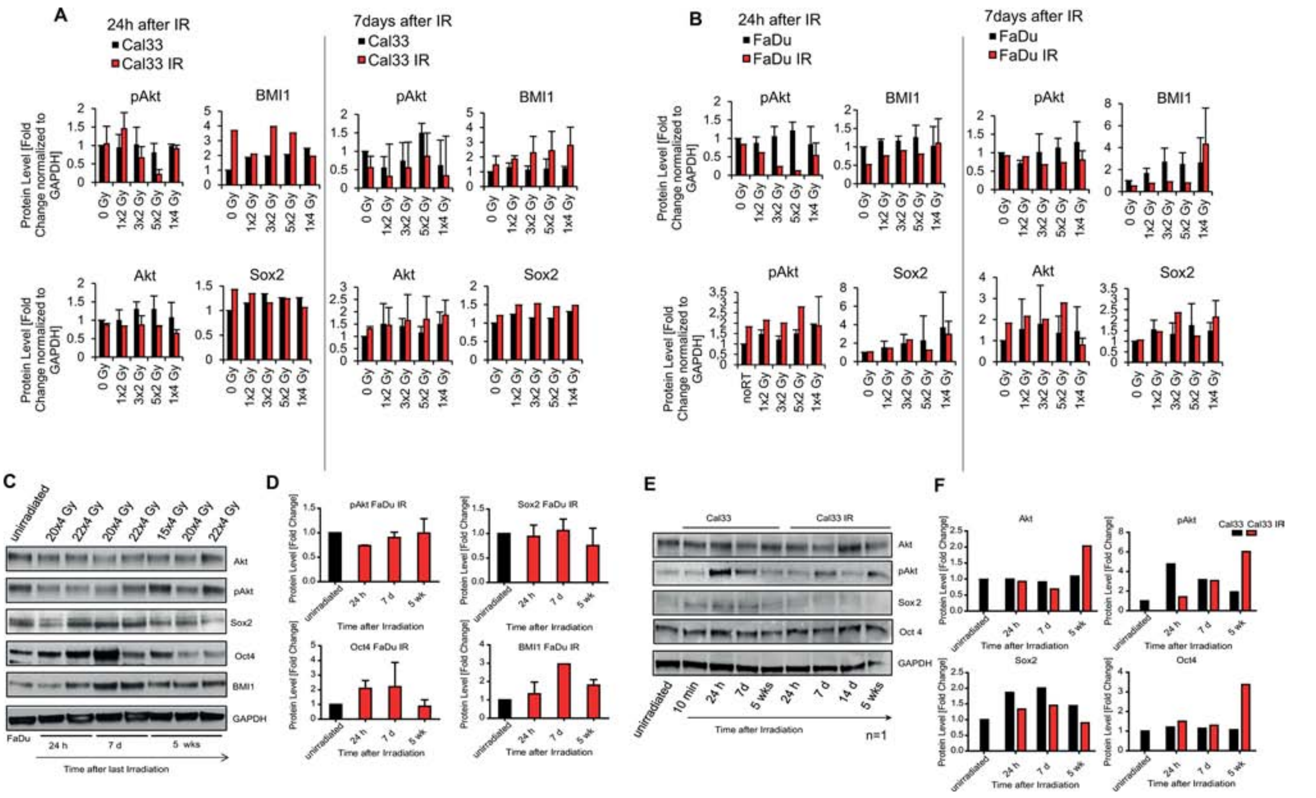

Supplementary Figure S3: CSC protein expression levels upon irradiation. A. quantification of the western blot from Figure 2B. **B.** quantification of the western blot from Figure 2C. **C.** Western blot analysis of FaDu IR subline analyzed at different timepoints after the last dose. **D.** quantification of the western blot from C. **E.** CSC protein changes upon irradiation in Cal333 and Cal33 IR. **F.** quantification of the western blot from F. (error bars = SD)

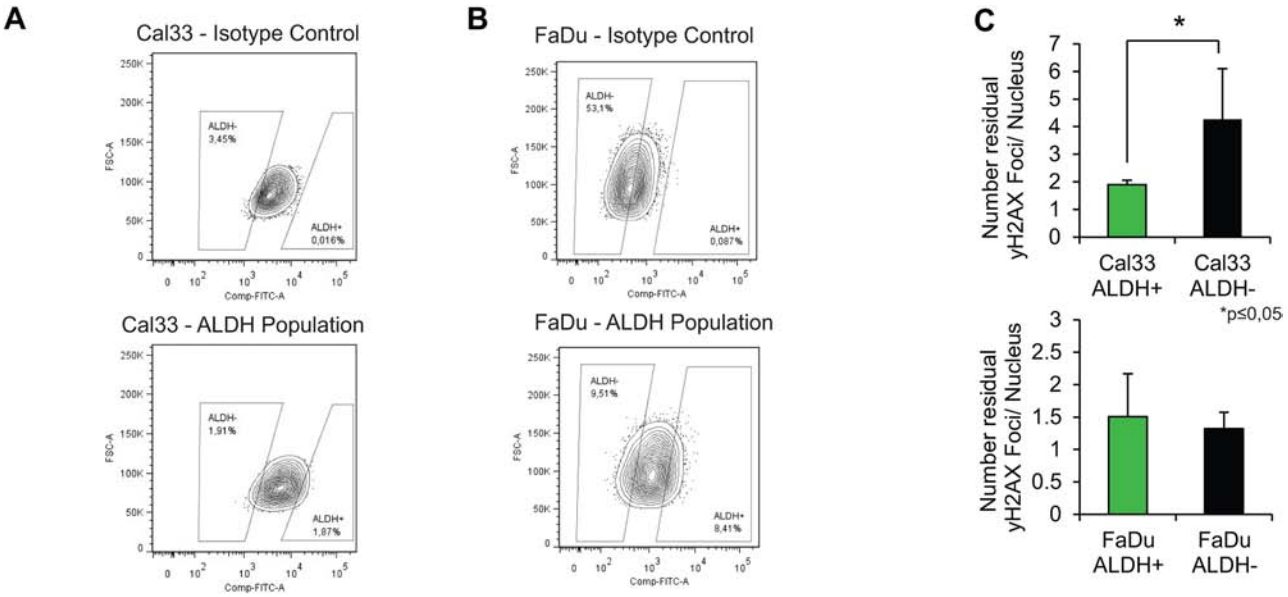

Supplementary Figure S4: ALDH as a biomarker for radioresistance. A. and **B.** Flow cytometric analysis of ALDH activity in Cal33 and FaDu **C.** absolute number of residual γH2AX foci formed after 4 Gy of X-ray irradiation for $ALDH^+$ and $ALDH^-$ FaDu and Cal33 HNSCC lines. ($p < 0,05$, error bars = SD).

A

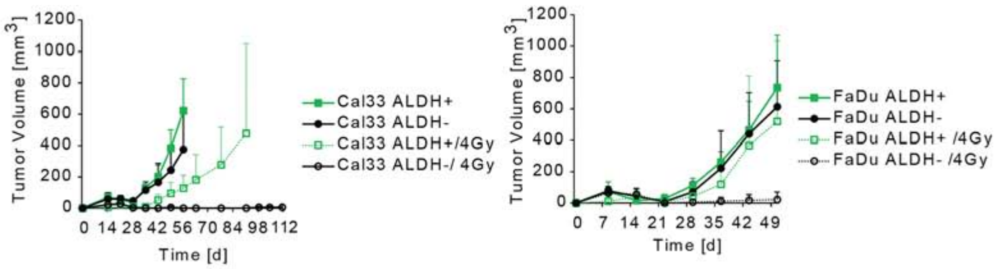

Time [d]	Cal33 ALDH+	Cal33 ALDH-	Cal33 ALDH+ /4Gy	Cal33 ALDH- /4Gy
0	0	0	0	0
14	0	0	0	0
21	0	0	0	0
28	0	0	0	0
35	5/5	3/5	0	0
42	5/5	3/5	0	0
49	5/5	5/5	2/5	0
56	5/5	5/5	2/5	0
63	†	†	3/5	0
77	†	†	4/5	0
91	†	†	4/5	0

Take rate: tumor diameter threshold = 100 µm

Time [d]	FaDu ALDH+	FaDu ALDH-	FaDu ALDH+ /4Gy	FaDu ALDH- /4Gy
0	0	0	0	0
8	0	0	0	0
15	0	0	0	0
22	0	0	0	0
29	2/4	1/4	1/5	0
36	4/4	3/4	2/5	0
43	4/4	4/4	3/5	0
50	4/4	4/4	3/5	0

Take rate: tumor diameter threshold = 100 µm

B

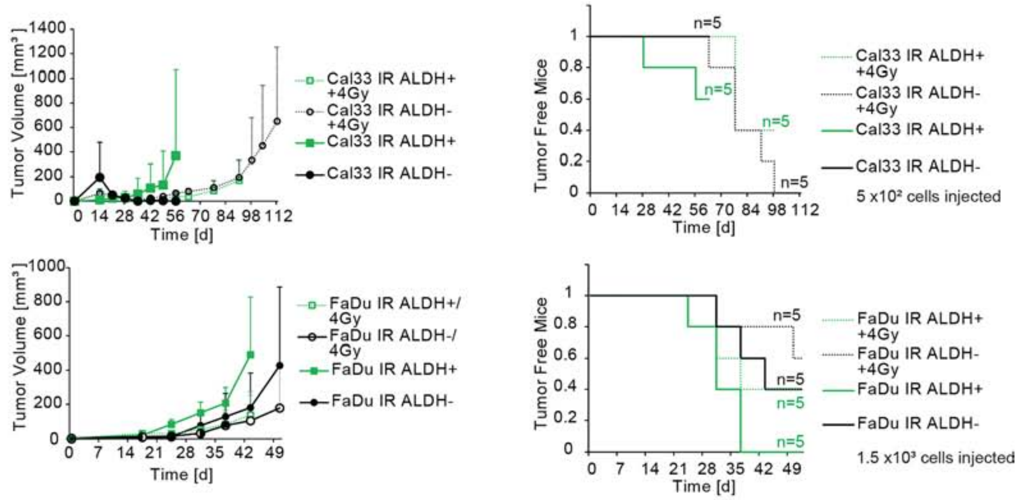

C

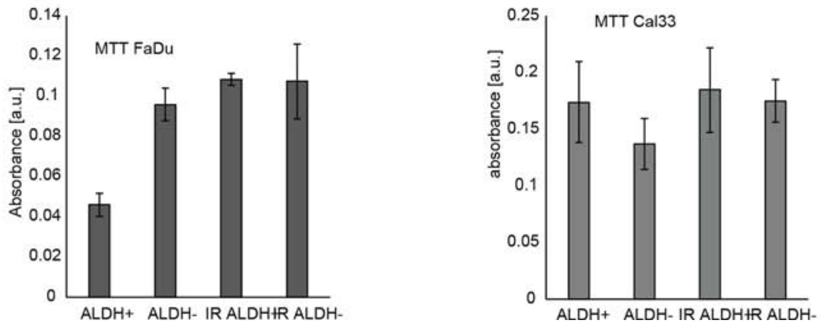

Supplementary Figure S5: Tumor formation of ALDH populations from Cal33 and FaDu. A. Tumorgrowth and up-take rates of Cal33 and FaDu ALDH^{+} and ALDH^{-} populations. **B.** Tumorgrowth Cal33 IR and FaDu IR ALDH^{+} and ALDH^{-} populations. **C.** Viability of ALDH sorted cells, which were injected into the mice. (error bars = SD).

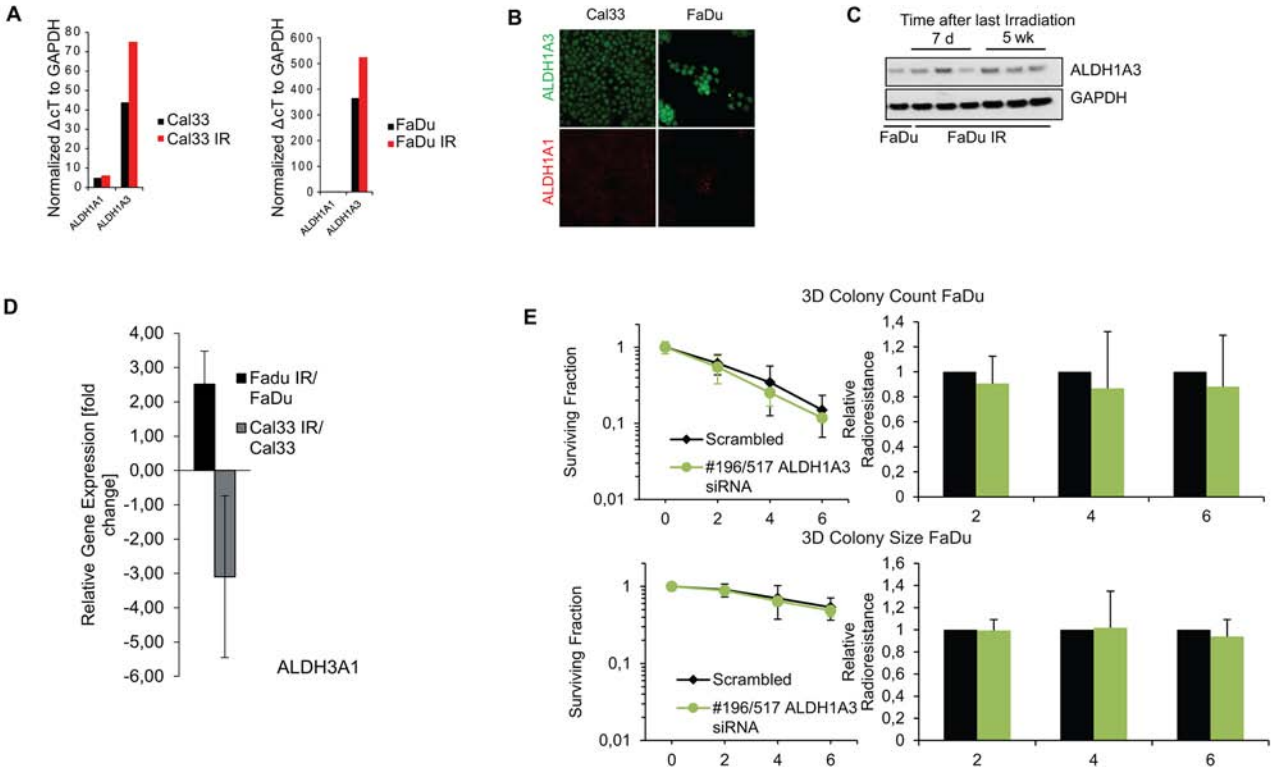

Supplementary Figure S6: Expression of ALDH1A3. A. RT PCR of ALDH1A3 RNA expression in Cal33, FaDu, and their respective IR sublines. **B.** immunfluorescent analysis ALDH1A1 and ALDH1A3 expression. **C.** Western blot analysis of ALDH1A3 in FaDu IR sublines. **D.** Analysis of gene expression of the ALDH3A1 isozyme. **E.** Reduction of the ALDH1A3 expression by siRNA results in a slight increase in cell radiosensitivity of FaDu cells as compared to the siRNA control cells. (n = 3, error bars = SD).
